# Dopaminergic PET to SPECT domain adaptation: a cycle GAN translation approach

**DOI:** 10.1007/s00259-024-06961-x

**Published:** 2024-11-19

**Authors:** Leonor Lopes, Fangyang Jiao, Song Xue, Thomas Pyka, Korbinian Krieger, Jingjie Ge, Qian Xu, Rachid Fahmi, Bruce Spottiswoode, Ahmed Soliman, Ralph Buchert, Matthias Brendel, Jimin Hong, Yihui Guan, Claudio L. A. Bassetti, Axel Rominger, Chuantao Zuo, Kuangyu Shi, Ping Wu

**Affiliations:** 1 Department of Nuclear Medicine, Inselspital, Bern University Hospital, University of Bern, Freiburgstrasse 18, Bern, 3010 Switzerland; 2 Graduate School for Cellular and Biomedical Sciences, University of Bern, Bern, Switzerland; 3 Department of Nuclear Medicine / PET Center, Huashan Hospital, Fudan University, Shanghai, 200235 China; 4 Department of Biomedical Imaging and Image-Guided Therapy, Division of Nuclear Medicine, Medical University of Vienna, Vienna, Austria; 5 TUM School of Medicine, Technical University of Munich, Munich, Germany; 6 National Center for Neurological Disorders & National Clinical Research Center for Aging and Medicine, Huashan Hospital, Fudan University, Shanghai, China; 7 Siemens Medical Solutions USA, Inc., Molecular Imaging, Knoxville, TN USA; 8 Department of Diagnostic and Interventional Radiology and Nuclear Medicine, University Medical Center Hamburg-Eppendorf, Hamburg, Germany; 9 Department of Nuclear Medicine, University Hospital, LMU Munich, Munich, Germany; 10 Munich Cluster for Systems Neurology (SyNergy), Munich, Germany; 11 German Center for Neurodegenerative Diseases (DZNE), Munich, Germany; 12 Department of Neurology, Inselspital, Bern University Hospital, University of Bern, Bern, Switzerland; 13 Department of Informatics, Technical University of Munich, Munich, Germany

**Keywords:** CFT PET, FP-CIT SPECT, Domain adaptation, Parkinson’s disease, Cycle GAN

## Abstract

**Purpose:**

Dopamine transporter imaging is routinely used in Parkinson’s disease (PD) and atypical parkinsonian syndromes (APS) diagnosis. While [^11^C]CFT PET is prevalent in Asia with a large APS database, Europe relies on [^123^I]FP-CIT SPECT with limited APS data. Our aim was to develop a deep learning-based method to convert [^11^C]CFT PET images to [^123^I]FP-CIT SPECT images, facilitating multicenter studies and overcoming data scarcity to promote Artificial Intelligence (AI) advancements.

**Methods:**

A CycleGAN was trained on [^11^C]CFT PET (*n* = 602, 72%PD) and [^123^I]FP-CIT SPECT (*n* = 1152, 85%PD) images from PD and non-parkinsonian control (NC) subjects. The model generated synthetic SPECT images from a real PET test set (*n* = 67, 75%PD). Synthetic images were quantitatively and visually evaluated.

**Results:**

Fréchet Inception Distance indicated higher similarity between synthetic and real SPECT than between synthetic SPECT and real PET. A deep learning classification model trained on synthetic SPECT achieved sensitivity of 97.2% and specificity of 90.0% on real SPECT images. Striatal specific binding ratios of synthetic SPECT were not significantly different from real SPECT. The striatal left-right differences and putamen binding ratio were significantly different only in the PD cohort. Real PET and real SPECT had higher contrast-to-noise ratio compared to synthetic SPECT. Visual grading analysis scores showed no significant differences between real and synthetic SPECT, although reduced diagnostic performance on synthetic images was observed.

**Conclusion:**

CycleGAN generated synthetic SPECT images visually indistinguishable from real ones and retained disease-specific information, demonstrating the feasibility of translating [^11^C]CFT PET to [^123^I]FP-CIT SPECT. This cross-modality synthesis could enhance further AI classification accuracy, supporting the diagnosis of PD and APS.

**Supplementary Information:**

The online version contains supplementary material available at 10.1007/s00259-024-06961-x.

## Background

Dopamine transporter (DAT) imaging, such as positron emission tomography (PET) with [^11^C]2β-carbomethoxy-3β-(4-fluorophenyl) tropane ([^11^C]CFT) and single photon emission computed tomography (SPECT) with [^123^I]2β-carbomethoxy-3β-(4-iodophenyl)-N-(3-fluoropropyl) nortropane ([^123^I]FP-CIT; DaTscan™, GE Healthcare), is a powerful tool in the differential diagnosis of idiopathic Parkinson’s disease (PD) from essential tremor or other secondary parkinsonism without nigrostriatal degeneration. However, this imaging tool is currently unreliable for differentiating PD from atypical neurodegenerative parkinsonian syndromes (APS), such as multiple system atrophy (MSA) or progressive supranuclear palsy (PSP) [1]. In clinical practice, the use of visual interpretation and semi-quantitative analysis has demonstrated high diagnostic accuracy [2–5] for PD and essential tremor differentiation. In research, semi-quantitative methods are generally preferred as they provide more objective measurements of DAT density [6]. The striatal specific binding ratio (SBR) is the most commonly used semi-quantitative measure [6, 7]. However, the lack of consistency in SBR measurements across different research sites, image acquisition techniques, reconstruction processes, and data analysis methods poses a challenge in longitudinal/multicenter studies. Recently, artificial intelligence (AI), higher, diagnostic accuracy than previous conventional methods [8], even in differentiating PD from APS [9, 10]. [^11^C]CFT PET is widely accessible in Asia and a substantial APS database was collected to support AI advancements [10]. [^123^I]FP-CIT SPECT is widely used in Europe [6, 11], however, limited APS data is available. A cross-modality synthesis between the two imaging techniques is appealing as it could allow better reproducibility of SBR and other quantitative measures and assist in AI diagnosis in modalities with a lack of sufficient data. Generative adversarial networks (GANs) [12] have remarkable capabilities in cross-modality medical image synthesis [13–17]. Moreover, GANs tackle various other medical challenges, such as image quality recovery [18] or CT-free PET paradigm [19]. These methods can alleviate data scarcity in medical research, by generating substantial quantities of realistic data. Cycle-consistency GAN (Cycle GAN) [20] stand out in medical image-to-image synthesis, as it does not need paired data for training, due to its cycle-consistency loss [21–24]. Cycle GAN has also been successfully used in multi-modality synthesis. In our case, although the same target is used (DAT), the modalities can differ due to different half-lives of the labels Carbon-11 and Iodine-123 and the different acquisition times. Thus, as we are dealing with different modalities and no paired data is available, we aimed to develop a Cycle GAN-based approach for cross-modality synthesis to improve interchangeability between [^11^C]CFT PET and [^123^I]FP-CIT SPECT.

## Materials and methods

### Data

This retrospective study included [^11^C]CFT PET brain images (DAT PET) from the Huashan Parkinsonian PET Imaging (HPPI) database and [^123^I]FP-CIT SPECT brain images (DAT SPECT) openly available from Parkinson’s Progression Markers Initiative (PPMI) database.

#### HPPI data

The Normal Controls (NC) cohort included 43 DAT PET scans from healthy subjects and 142 from subjects with normal DAT imaging, with a total of 185 subjects. In the PD cohort, we included 484 DAT PET from patients diagnosed with PD.

All included patients from the HPPI performed a DAT PET and an MRI to exclude structural brain abnormalities at Huashan Hospital. Patients with PD were diagnosed by movement disorder specialists on their return visits after PET examination, according to the current diagnostic criteria [25]. The inclusion criteria for healthy controls and normal DAT subjects can be found in the supplementary materials.

#### DAT PET acquisition and reconstruction

DAT PET scans were were all acquired with a Biograph™ 64 HD PET/CT (Siemens Medical Solutions USA, Inc., Molecular Imaging, Knoxville, TN), one hour after an intravenous injection of 333–407 MBq (9-11mCi) of [^11^C]CFT. The duration of acquisition was 15 min. Low dose CT was performed previously for attenuation correction. Iterative 3D-ordered subset expectation maximization algorithm was used to reconstruct the images after corrections for scatter, dead time and random coincidences.

#### PPMI data

The PPMI is a large, international multicenter clinical study that aims to identify various biomarkers for the progression of de novo PD.

We included 194 reconstructed [^123^I]FP-CIT SPECT (DaTscan™) scans from the Healthy Controls Cohort– NC group– and 1086 from the PD cohort– PD group– from the PPMI initiative (www.ppmi-info.org/data).

Participants in this study were individuals diagnosed with PD who were at least 30 years old, regardless of gender. The PPMI study had specific criteria for participant eligibility that can be found at https://www.ppmi-info.org/study-design/research-documents-and-sops.

#### DAT SPECT acquisition and reconstruction

DAT SPECT scans are acquired four hours after injection of 3–5 mCi (111–185 MBq) of DaTscan™. CT was performed for attenuation correction and the Hermes (Hermes Medical Solutions, Stockholm) iterative ordered-subsets-expectation-maximization algorithm was used to reconstruct the images. The detailed PPMI [^123^I]FP-CIT SPECT protocol can be found at https://www.ppmi-info.org/study-design/research-documents-and-sops.

### Image preprocessing

Before inputting the images into the model, they were spatially normalized into the Montreal Neurological Institute (MNI) brain space using SPM 5 (https://www.fil.ion.ucl.ac.uk/spm), implemented in Matlab 7.4.0 (Mathworks Inc, Sherborn, MA). To facilitate the Cycle GAN training the images were then smoothed by a 3D Gaussian filter of 10 mm for PET and 6 mm for SPECT full width at half maximum (FWHM). Intensity normalization was performed by dividing each voxel by the maximum value of each training dataset (described below). We applied the SPM brain mask from the MNI brain space atlas before inputting the images into the CycleGAN.

### Cycle GAN

#### Model

A 3D CycleGAN [20] was developed to make the image-to-image translation between two domains, DAT PET imaging and DAT SPECT imaging. The CycleGAN model includes two generators (G_PS_ - PET to SPECT- and G_SP_– SPECT to PET) and two associated adversarial discriminators (D_P_ and D_S_). Each of the discriminators encourages its corresponding generator to synthesize images similar to the original ones by minimizing an adversarial loss function. The synthesized SPECT images are then translated back to the original PET domain, with the G_SP_ (and vice-versa for the synthetic PET images). The cycle consistency loss helps ensure that the translated images are similar to the real ones. The PET scan is inputted into the G_SP_ and vice-versa (SPECT to the G_PS_) and the output image is compared to the real PET (and SPECT) image, through the identity loss.

**Fig. 1:**
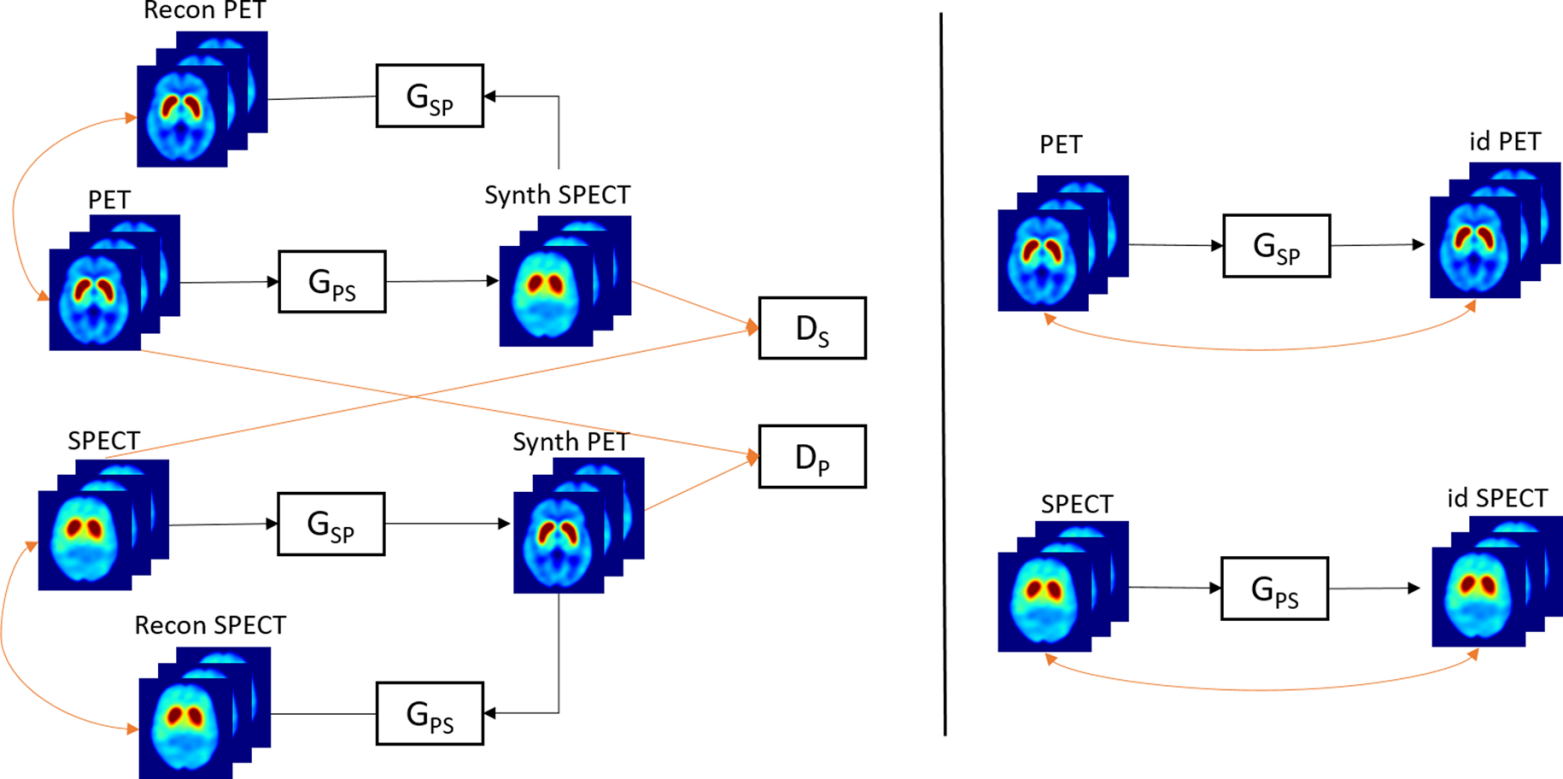
Scheme of our Cycle GAN model. G_PS_: Generator of PET to SPECT; G_SP_: Generator of SPECT to PET. D_P_: Discriminator of real and synthetic PET; D_S_: Discriminator of real and synthetic SPECT; Recon PET/SPECT: Reconstructed PET/SPECT back to original domain; Synth PET/SPECT: Synthetic PET/SPECT; id PET/SPECT: identity PET/SPECT that should be unchanged when passed through the G_SP_/G_PS_ generators

Detailed formulas and networks structures are shown in the supplementary material. Figure 1 shows a scheme of our cycle GAN model.

#### Training and image generation

We trained the CycleGAN with 90% of PPMI SPECT and HPPI PET and used the remaining 10% as testing datasets to evaluate the performance of the model. For these evaluations, we generated synthetic SPECT images from the real PET images in the testing dataset. We applied the SPM brain mask in the MNI space and a Gaussian filter of 1.7 FWHM before further analysis, to smooth the images with a grid-like texture (Supplementary Fig. 2). Table 1 shows the number of scans from each dataset in training and testing procedures.

The model was trained in a NVIDIA GeForce RTX 2080 Ti GPU (NVIDIA Corporation, Santa Clara, CA, USA) for 200 epochs with a batch size of 1 due to memory constrains. We used binary cross entropy loss for the adversarial loss and L1 loss for the cycle consistency and identity losses, with weights of 10 for the cycle consistency and 1 for the others. Adam optimizer was used with a learning rate of 2e-4.

**Table 1:** Huashan Parkinsonian PET Imaging (HPPI) and Parkinson’s progression markers Initiative (PPMI) datasets’ size and corresponding training and testing splits’ size per class/disease

		HPPI PET data	PPMI SPECT data
Training dataset	NC	168	174
	PD	434	978
Testing dataset	NC	17	20
	PD	50	108
Total		669	1280

NC: Normal controls; PD: Parkinson disease

### Evaluation of results

#### Fréchet inception distance

To assess the high-level perceptual image similarity between synthetic SPECT and real SPECT and PET, we calculated the Fréchet Inception Distance (FID). The FID, introduced by Heusel, M. et al. in 2017, is a widely adopted metric to compare the quality of images synthesized by generative models, particularly when paired data is not available and thus, methods such as root-mean-square error (RMSE) and structural similarity index (SSIM) cannot be used. A lower FID value suggests greater similarity between the two datasets in terms of their statistical properties. FID is calculated as the Wasserstein-2 distance between the multi-variate Gaussians fitted to data embedded into a feature space, employing a pre-trained Inception V3 network. We performed bootstrapping with replacement to perform statistical comparison (Student’s t-test) between FIDs of synthetic SPECT with real SPECT and synthetic SPECT with real PET.

#### Deep-learning classification model

A classification network was trained using the synthetic SPECT data and tested on the real SPECT test set. A previously developed network, based on the ResNet architecture and validated for the differential diagnosis between PD and APS, was used [10]. The final linear layer of this network was modified to enable binary classification to differentiate between two classes (NC and PD) with log sigmoid activation. The highest log probability (NC or PD) determined the prediction for diagnostic evaluation. The model was trained for 50 epochs, with early stop if validation accuracy did not improve in 30 epochs. The initial learning rate was 1e-4 and was reduced by a factor of 0.5 when validation loss did not improve for 10 epochs. Adam optimizer and the negative log likelihood loss were used.

#### Semi-quantitative analysis

We calculated the striatum specific binding ratio (SBR), caudate specific binding ratio (CBR) and putamen specific binding ratio (PBR) for each already preprocessed image in the synthetic SPECT, real SPECT, and real PET test datasets and then assessed the differences between test datasets. The regions of interest (ROI), including striatum, putamen or caudate, binding ratios were calculated as follows:

$$\:\frac{mean\:counts\:of\:ROI-mean\:counts\:of\:background\:region}{mean\:counts\:of\:background\:region},$$

where the background region corresponds to a region within the occipital lobe.

The ROI and occipital regions were obtained by applying the CerebrA template to each image after being registered to the MNI space [26].

We also calculate the absolute differences between left (SBR_L_) and right striatum (SBR_R_) as $\:\left|{\text{S}\text{B}\text{R}}_{L}-\:{\text{S}\text{B}\text{R}}_{R}\:\right|.$

#### Contrast-to-noise ratio

To measure the image quality, we also calculated the contrast-to-noise ratio (CNR) for each already preprocessed image in the synthetic SPECT, real SPECT, and real PET test datasets and then assessed the differences between test datasets. The CNR was calculated as follows:

$$\begin{aligned}&CNR\cr&\quad=\frac{\begin{aligned}&mean\:counts\:of\:striatum\\&-mean\:counts\:of\:background\:region\end{aligned}}{standard\:deviation\:of\:counts\:of\:background\:region}\end{aligned}$$

#### Blind visual assessment

Four nuclear medicine physicians (T.P.: 5 years of experience; K.K.: 2 years of experience; J.G.: 6 years of experience F.J.: 7 years of experience) evaluated the quality of the synthetic images through absolute visual grading analysis (VGA) [27]. Ten synthetic SPECT images were generated from 10 real PET (5 NC and 5 PD) randomly selected from the HPPI test set. Additionally, 10 real SPECT images from the PPMI test set were chosen randomly. These 20 datasets were mixed and presented to the readers, who had no information about data source (synthetic or original) or diagnosis (NC or PD).

Readers, untrained in detecting synthetic images, assessed images based on four criteria using a 3-point Likert scale: level of noise (1 = low, 2 = medium, 3 = high), presence of artifacts (1 = absent, 2 = uncertain, 3 = present), synthetic appearance (1 = real, 2 = uncertain, 3 = synthetic), and confidence in diagnosis (1 = insufficient, 2 = sufficient, 3 = good/confident). VGA Scores (VGAS) for each of these four criteria were computed for each image dataset (real and synthetic SPECT) based on these assessments as:

$$\:VGAS=\:\frac{\sum_{O,\:I\:}{S}_{c}}{{N}_{i\:}{N}_{o}},$$

where $\:{S}_{c}$ are the given individual scores for observer (O) and image (I), $\:{N}_{i\:}$is the total number of images and $\:{N}_{o}$ is the total number of observers.

The physicians also made a diagnosis, as either NC or PD.

### Statistical analysis

Statistical analysis was performed using *Python* 3.11.4 with the libraries *Scipy* 1.11.1 and *Scikit-learn* 1.3.0. Cycle GAN was implemented using *Keras* 2.12.0 library and the DL classification model using *Pytorch* 1.13.0. Differences were considered significant for *p* < 0.05, using two-sided p-values. Effect size was calculated with Cohen’s d. Shapiro-Wilk test was used to assess sample normality, and statistical tests chosen accordingly. Differences in FID, SBR, CBR, PBR values and left-right striatum differences were assessed by the Student’s *t*-test and p-values and 95% confidence intervals (95%CI) are shown. Values are presented as mean ± standard deviation (SD). The area under the receiver operating characteristic (ROC) curve (AUC), sensitivity, specificity, positive predictive value (PPV), and negative predictive value (NPV) were obtained to evaluate the performance of the classification DL model. Differences in CNR were assessed by Mann-Whitney U test. CNR are presented as mean ± SD. Differences in VGAS were assessed by the Mann-Whitney U test. VGAS were presented as mean ± SD.

## Results

### Fréchet inception distance

The FID between real PET and synthetic SPECT test sets (152.3 ± 0.58) was higher than the FID between real SPECT and synthetic SPECT (142.6 ± 0.70; *p* < 0.001; 95%CI [-9.78, -9.53]).

### Deep-learning classification

**Fig. 2:**
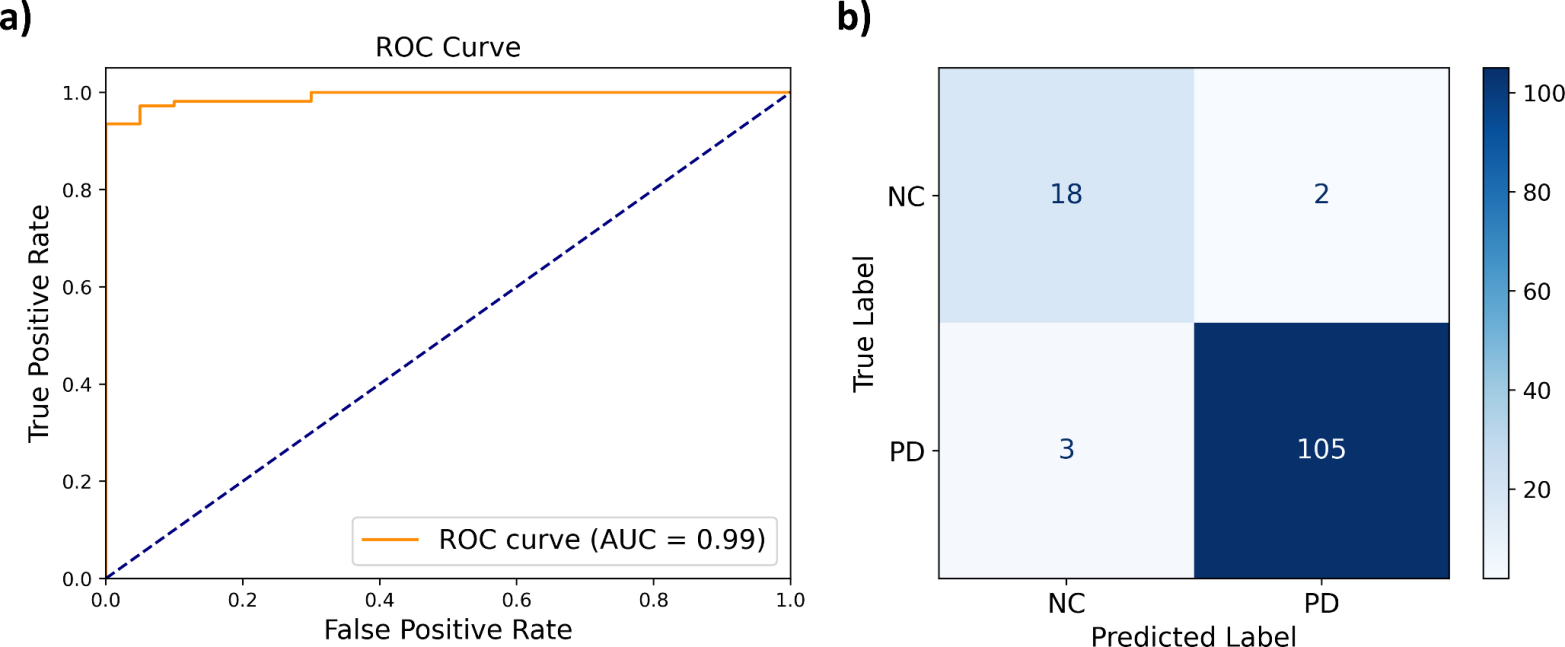
Performance of the DL classification model in NC vs. PD classification in the real SPECT test set. (**a**) Receiver Operating Characteristics (ROC) Curve. (**b**) Confusion Matrix. NC: Normal controls; PD: Parkinson disease

The ROC curve of the DL classification model is presented in Fig. 2. The model trained with synthetic SPECT achieved an AUC of 0.992, sensitivity of 97.2%, specificity of 90.0%, PPV of 98.1%, and NPV of 85.7% for the classification of the real SPECT test set into NC vs. PD.

### Semi-quantitative analysis

**Fig. 3:**
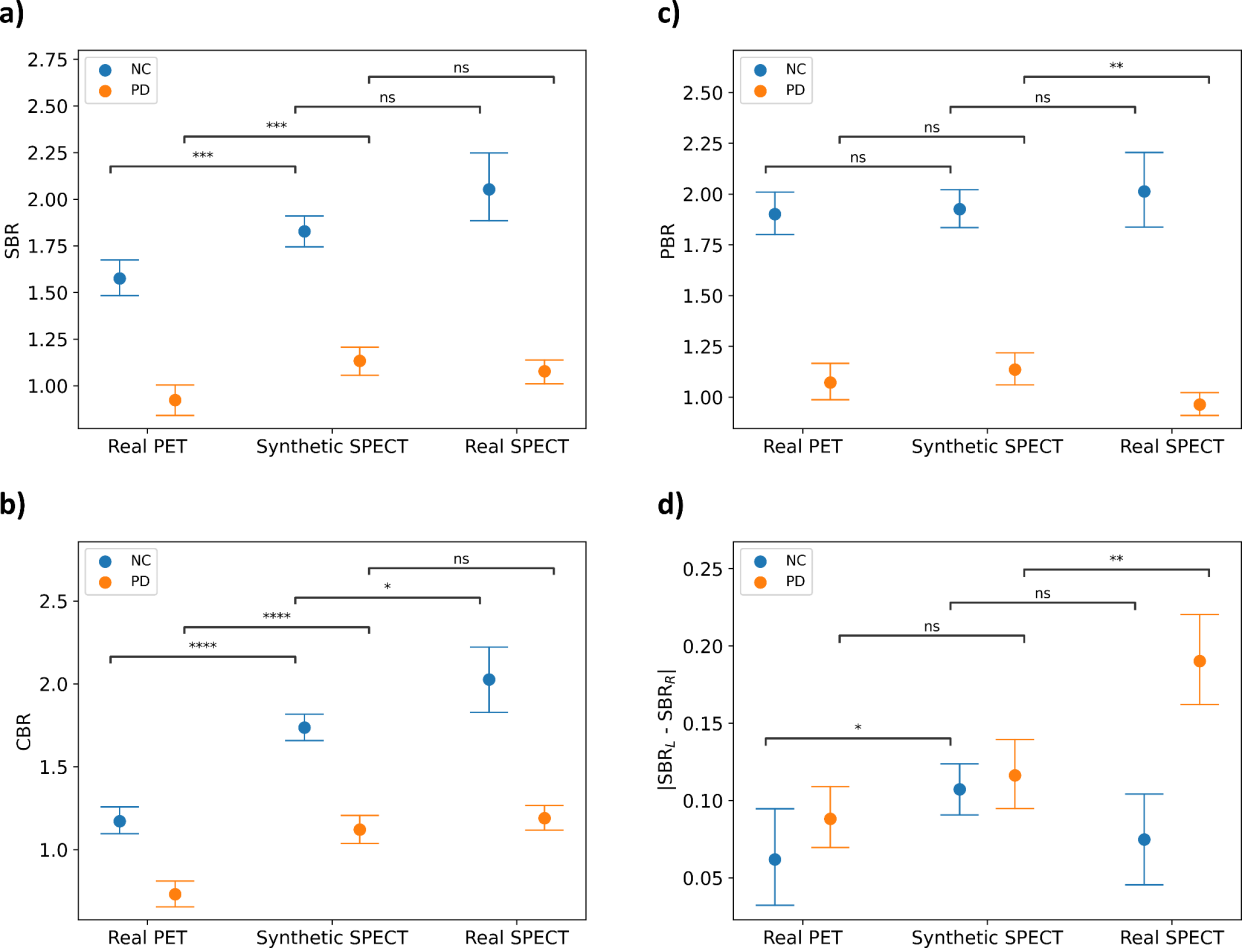
Average Specific Binding Ratios of the synthetic SPECT, real SPECT, and real PET test datasets, both in NC and PD, for striatum (**a**), caudate (**b**) and putamen (**c**) and left-right striatal differences (**d**). Error bars represent the standard deviation. ns: non-significant, *p < 0.05, **p < 0.01, ***p < 0.001, ****p < 0.0001 (Student’s *t*-test). NC: Normal controls; PD: Parkinson disease

As shown in Fig. 3a, the SBR values of the NC synthetic SPECT dataset (1.83 ± 0.17) were not significantly different from the SBRs of the NC real SPECT (2.05 ± 0.42; *p* = 0.05, 95%CI [0.00, 0.45]). Similar results were found for the PD synthetic SPECT (1.13 ± 0.29) versus the real SPECT dataset (1.10 ± 0.33; *p* = 0.31, 95%CI [-0.05, 0.16]). However, differences were significant (*p* < 0.001) between synthetic SPECT and real PET (NC: 1.57 ± 0.20; 95%CI [0.12, 0.39]; PD: 0.92 ± 0.30; 95%CI [0.09, 0.33]), in both NC and PD. Similarly, to real PET and SPECT data, significant differences were found in striatal SBR in synthetic SPECT between NC and PD groups (*p* < 0.001; 95%CI [0.54, 0.84]).

In Fig. 3b, the caudate binding ratios (CBRs) of the NC synthetic SPECT dataset (1.74 ± 0.17) were significantly different from the CBRs of the NC real SPECT (2.02 ± 0.46; *p* = 0.02; 95%CI [0.05, 0.54]), with smaller differences than when comparing to NC real PET (1.17 ± 0.17; *p* < 0.001; 95%CI [0.44, 0.69]). In the PD cohort, the CBRs were not significantly different in the synthetic SPECT (1.12 ± 0.31) compared to the real SPECT (1.19 ± 0.38; *p* = 0.26; 95%CI [-0.19, 0.05]). Significant differences were found in CBRs in synthetic SPECT between NC and PD groups (*p* < 0.001; 95%CI [0.46, 0.77]).

In Fig. 3c, the putamen binding ratios (PBRs) differences are significant in the PD cohort between synthetic SPECT (1.14 ± 0.27) and real SPECT (0.96 ± 0.31; *p* = 0.001; 95%CI [0.07, 0.27]). PBR differences in the synthetic SPECT NC (1.93 ± 0.18) and PD cohorts are significant (*p* < 0.001; 95%CI [0.65, 0.93]).

As shown in Fig. 3d, absolute differences between right and left SBR are significant between PD synthetic SPECT (0.11 ± 0.09) and PD real SPECT (0.19 ± 0.15; *p* = 0.001; 95%CI [-0.12, -0.03]) and between NC synthetic SPET (0.11 ± 0.03) and NC real PET (0.07 ± 0.07; *p* = 0.02; 95%CI [0.01, 0.08]).

### Contrast-to-noise ratio

**Fig. 4:**
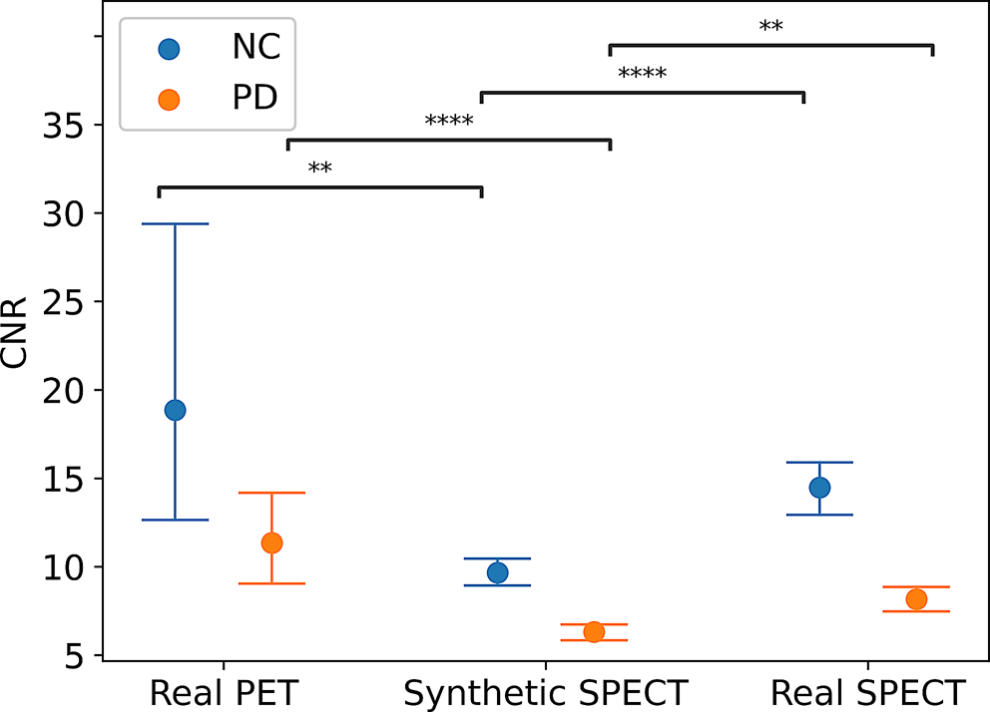
Average contrast-to-noise (CNR) of the synthetic SPECT, real SPECT, and real PET test datasets, both in NC and PD. Error bars represent the standard deviation. **p < 0.01, ****p < 0.0001 (Mann-Whitney U test). NC: Normal controls; PD: Parkinson disease

The CNR was higher in real PET of both NC (18.85 ± 17.56) and PD (11.34 ± 9.74) compared to Synthetic SPECT of NC (9.66 ± 1.64; *p* = 0.002) and PD (6.31 ± 1.67; *p* < 0.001), respectively. The CNR was also higher in NC (14.48 ± 3.49) and PD (8.16 ± 3.62) of real SPECT compared to NC (*p* < 0.001) and PD (*p* = 0.002) of Synthetic SPECT. Results are presented in Fig. 4.

### Blind visual assessment

**Fig. 5:**
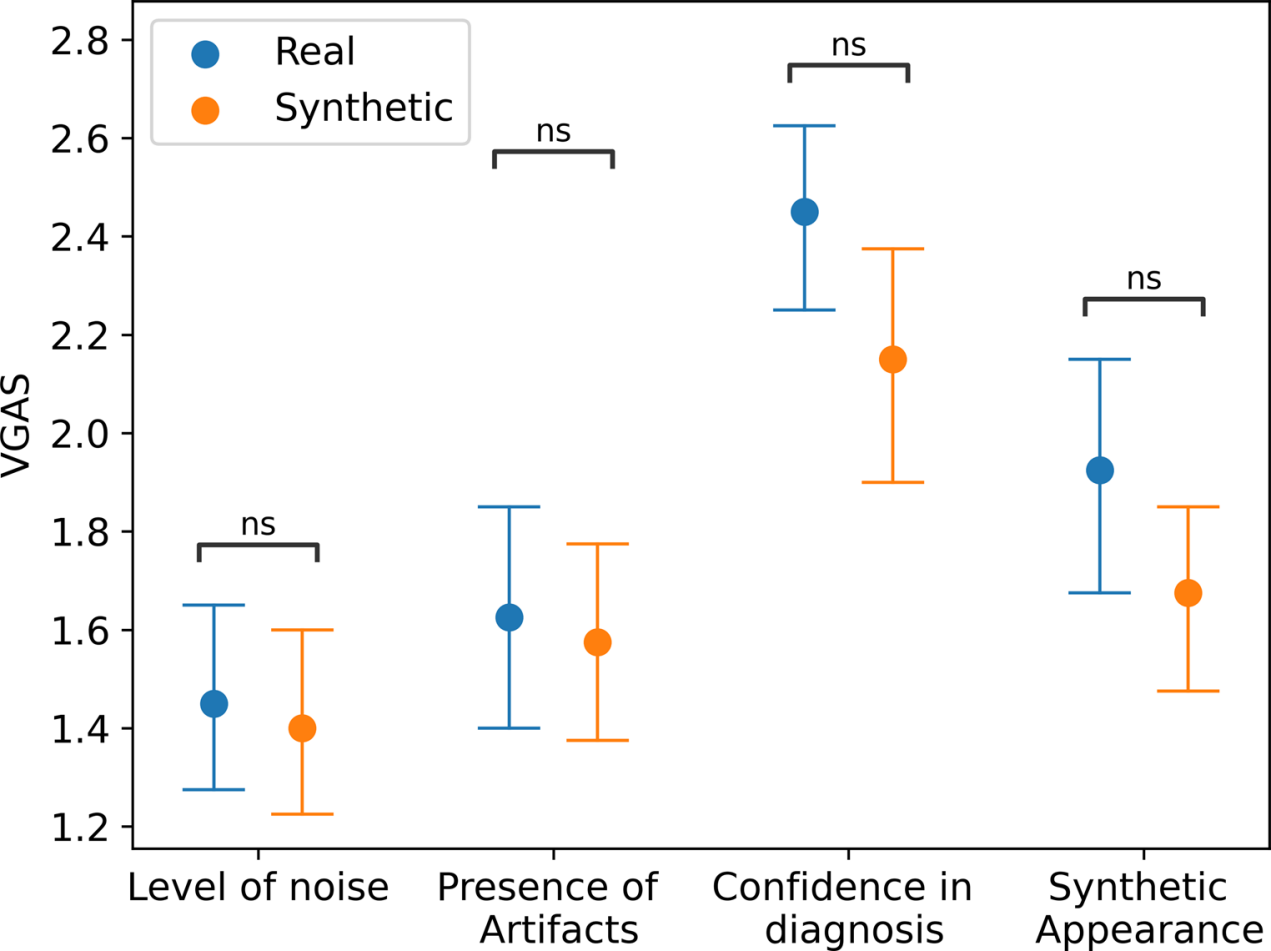
Differences in Visual Grading Analysis Score (VGAS) between real and synthetic SPECT images in all the categories analyzed– level of noise, presence of artifacts, confidence in diagnosis, and synthetic appearance. Error bars represent the standard deviation. ns: *p* > 0.05 (Mann-Whitney U test)

As shown in Fig. 5, Mann-Whitney U test showed no significant differences between real and synthetic SPECT in VGAS of all criteria analyzed (Synthetic Appearance: *p* = 0.18; Level of Noise: *p* = 0.73; Artifacts: *p* = 0.86; Confidence in diagnosis: *p* = 0.11). The results of individual readers are presented in the Supplementary Table 1.

As displayed in Table 2, the NC vs. PD diagnostic performance of the majority of the readers is lower in synthetic SPECT images than in the real ones.

**Table 2:** Diagnostic performance in terms of AUC, sensitivity, specificity, PPV, and NPV of individual readers on real and synthetic SPECT images. Diagnostic performance corresponds to the ability to differentiate NC vs. PD cases

Reader	Dataset	AUC	Sensitivity (%)	Specificity (%)	PPV (%)	NPV (%)
#1	Real	0.90	100	80	83.3	100
	Synthetic	0.90	100	80	83.3	100
#2	Real	0.90	100	80	83.3	100
	Synthetic	0.80	60	100	100	71.4
#3	Real	0.70	100	40	62.5	100
	Synthetic	0.50	80	20	50	50
#4	Real	0.90	100	80	83.3	100
	Synthetic	0.60	40	80	66.6	57.1

.

**Fig. 6:**
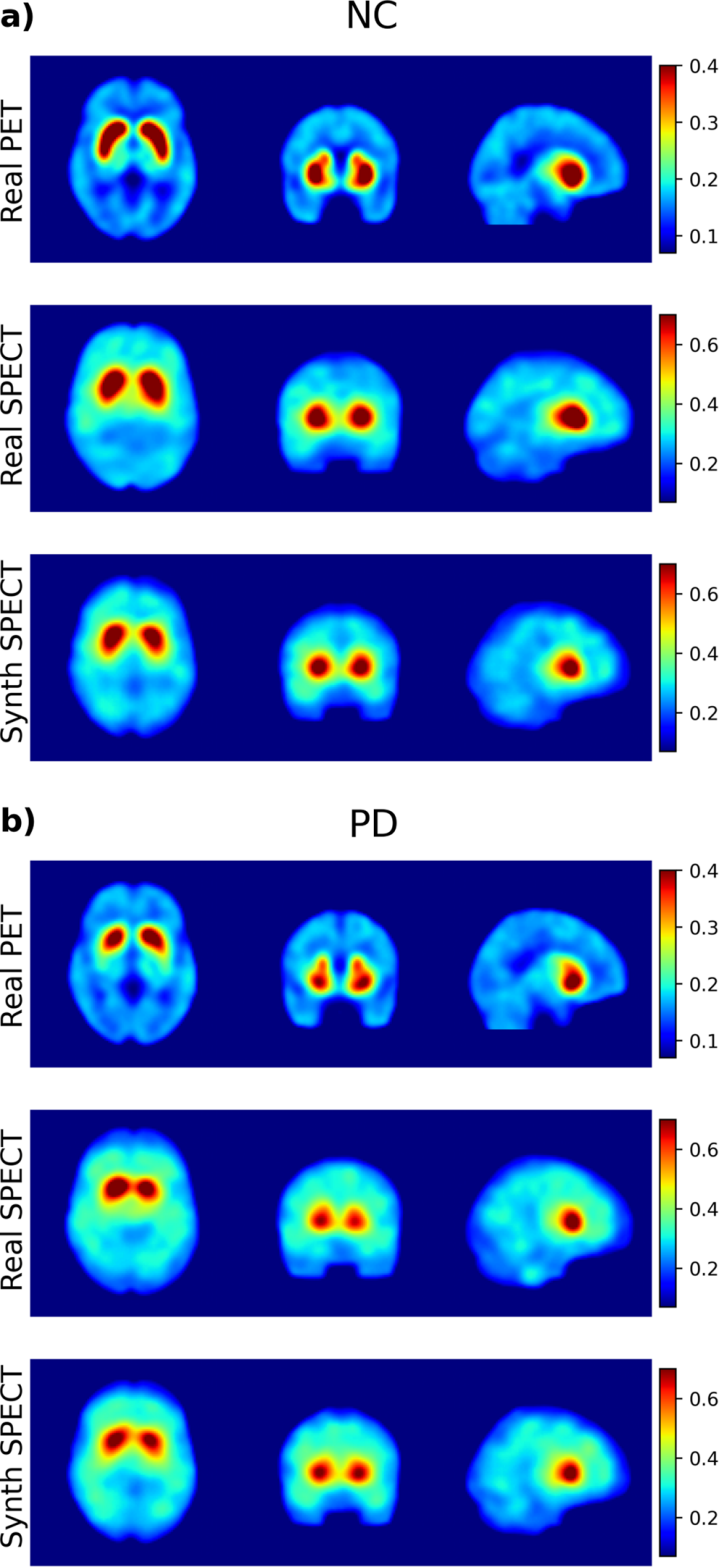
Example of images of (**a**) normal controls and (**b**) Parkinson’s disease from real PET (upper row), real SPECT (middle row) and synthetic SPECT (bottom row)

Examples of 2 cases of real and synthetic SPECT images from both NC and PD groups are shown in Fig. 6. Mean images are shown on Supplementary Fig. 1. An example of a synthetic SPECT with artifacts is shown in Supplementary Fig. 3.

## Discussion

In this study, we explored for the first time the domain adaptation from DAT PET to DAT SPECT in the context of PD. Our results demonstrated the potential of Cycle GAN as a valuable tool for bridging the domain gap between these two modalities, improving AI-based diagnosis.

One of the key achievements of this study was the generation of visually indistinguishable synthetic DAT SPECT images from the DAT PET images. The FID of synthetic SPECT images compared to real SPECT was lower than that of synthetic SPECT compared to real PET. This suggests that the synthetic SPECT images closely resemble real SPECT not only in visual appearance but also in high-level statistical features. This finding highlights the effectiveness of our approach in preserving essential characteristics of the original SPECT images during the translation process.

Furthermore, the deep learning classification model trained on these synthetic images demonstrated strong performance in identifying PD in real SPECT data. This result highlights the potential of Cycle GAN in expanding the availability of training data for DL models, thereby reducing the dependency on large, expensive datasets. The synthetic images maintained the necessary functional information for DL classification, showing that our approach could be a valuable tool in data-scarce environments, particularly for multi-center studies involving different imaging modalities. As no real MSA or PSP SPECT data was available and, therefore, no tests could be performed on these data kinds, their synthetic counterparts were not generated.

At a semi-quantitative level, SBRs of synthetic DAT SPECT were not significantly different from the ones of the real DAT SPECT. Additionally, lower SBR values were found for the synthetic PD compared to synthetic NC images, confirming the preservation of disease-specific information in the image-to-image translation. SBR is a quantitative measure used in research and complements visual readings in clinical practice [5, 28–30]. However, the SBR values from different datasets are not consistent, due to subject-related physiological factors and different imaging systems, including different acquisition hardware and different image reconstruction software [31–33]. Thus, it complicates comparisons in multicenter/longitudinal studies. Other alternatives, such as phantom-based calibration and dedicated reconstruction algorithms attempted to mitigate this variability [33], but these approaches are not straightforward, as they involve addressing camera-specific factors. In our case, we have not just different subject populations, cameras, centers, but also different imaging modalities. As DAT SPECT is most used in European centers/studies [6, 11] and DAT PET is widely used in Asia, studies using both of these modalities can be of major importance, especially due to the limited APS data. Because Cycle GAN performed well in these different modality cases, we foresee that it can be used in simpler multi-system pipelines. However, there remain some areas for improvement. In the PD cohort, differences were noted in the putamen binding ratio and left-right striatal differences between synthetic and real SPECT, indicating that while the translation process preserves crucial diagnostic features, (such as decreased SBR, PBR and CBR in the PD cohort compared to NC) it is not yet perfect. Additionally, in terms of contrast-to-noise ratio, the synthetic SPECT have lower values than both real SPECT and synthetic SPECT, meaning a decrease in terms of contrast of striatum.

Blind visual assessment by the nuclear medicine physicians confirmed structural similarity between real and synthetic SPECT, with no differences in VGAS of analyzed categories (synthetic appearance, level of noise, presence of artifacts, and confidence in diagnosis). However, 3/4 physicians had reduced diagnostic accuracy in synthetic images compared to real ones, possibly due to the attenuation of certain discriminative features during translation, enough for DL network to identify, but not the physicians. This is also consistent with the differences in the putamen binding ratio and the left-right striatal differences, as well as with the low CNR.

When comparing Cycle GAN to other domain adaptation methods like AttentionGAN and diffusion models, several differences emerge. AttentionGAN [34] leverages attention mechanisms to enhance feature preservation, potentially offering more precise image translations but at the cost of increased complexity and computational demand. Diffusion models [16, 35, 36] are known for generating high-quality images through iterative noise reduction but require extensive computational resources and long training times. In contrast, Cycle GAN strikes a balance between image quality and computational efficiency, making it more practical for medical imaging applications. However, it may struggle with fine-grained feature preservation, as indicated by the subtle discrepancies observed in our study. While Cycle GAN is effective for many tasks, integrating aspects of these advanced methods could further enhance translation quality in future research.

In terms of clinical relevance, our method could significantly enhance AI-assisted diagnostic tools by increasing the availability of training data, thereby making these tools more robust and generalizable. Several studies show that integrating deep learning-based data augmentation is an important strategy in this regard [17]. It could also provide additional resources for educating new physicians. Furthermore, the ability to translate PET to SPECT could facilitate clinical comparisons of patient scans—where PET imaging was used—with existing standard databases of normal controls, which are predominantly SPECT-based in Europe [31]. However, for clinical adoption, it will be essential to address challenges such as meeting regulatory standards for synthetic images, integrating the method into existing clinical workflows, and ensuring the interpretability and reliability of AI-generated images.

It is important to acknowledge some other limitations. While our study focused on generating SPECT images from PET images due to current needs, generating PET images from SPECT could be a more advantageous approach given the higher spatial resolution of PET. However, translating from SPECT to PET presents additional challenges, especially with only limited and unpaired data are available. Future studies should consider exploring this approach to leverage the higher detail in PET images.

Although Cycle GAN has shown promise in domain adaptation for medical imaging [37, 38], challenges like data heterogeneity and the need for large-scale datasets for robust training still remain [20, 39, 40]. A common issue in synthetic image generation is hallucination, where the model generates artifacts in the synthetic image [41]. However, since our main goals is to enhance DL classification, minor structural abnormalities in synthetic images are less problematic. Additionally, tuning the parameters and the training approach proved to be challenging, as minor adjustments in the hyperparameters affect training stability [39, 42]. An external dataset for testing would be a valuable addition in future studies, to improve the robustness and generalizability of our results.

In the image quality quantitative metrics, CNR is low especially on the NC cohort, which can impair visual analysis. Nevertheless, SBR are similar in the synthetic and real SPECT and DL classification has high accuracy, meaning that even if not ideal for visual assessment, the synthetic images are helpful for semi-quantitative and DL-based diagnosis. It is also noteworthy that the SBR difference between real SPECT and synthetic SPECT is bigger in the NC that in the PD, even though not significantly. This can be due to the unbalanced training dataset, which holds much more PD (80%) than NC (20%). A more balanced training set could improve this disparity. Another limitation is the use of Normal DAT imaging scans and not just NC and the different PD/NC proportions for PET and SPECT datasets.

In the visual assessment, there are also important limitations. The interpretation and generalization of the results can be limited by the small sample size (*n* = 20) and the Gaussian filters applied. As the spatial resolution of the PPMI SPECT images is already low, further reduction might have an impact on the diagnostic utility. Nonetheless, diagnostic performance of the four readers on real SPECT images was good (AUC > 0.70). Moreover, some studies show that domain adaptation might be achieved by simply smoothing images [43]. Further studies should aim for larger samples and fewer preprocessing steps.

Lastly, the impact of domain adaptation on downstream tasks, such as disease classification, should be thoroughly investigated. Although quantitative metrics such as SBR and CNR offer some insight into the translation process and synthetic SPECT images appear realistic, they do not allow for direct evaluation of the synthetic images. We cannot know how comparable a real clinical SPECT image would be to the corresponding synthetic image, due to the absence of paired data. It is essential that next studies include paired datasets – as a more accurate and direct way to evaluate the synthetic images – and assess how well the synthetic data can improve the performance of existing AI models and clinical decision support systems in the context of DAT imaging.

## Conclusion

Our study highlights the potential of Cycle GAN in DAT PET to DAT SPECT domain adaptation. This approach holds promise for more multicenter/longitudinal comparison studies and for expanding data availability needed to enhance the accuracy in diagnosing parkinsonian disorders. Future research is needed to address the remaining challenges and evaluate the clinical applicability of the proposed approach.

## Electronic supplementary material

Below is the link to the electronic supplementary material.

Supplementary Material 1

## Data Availability

The datasets generated and analyzed during the current study are available from the corresponding author upon reasonable request. Our code is available for download at https://github.com/lopes-leonor/DAT-cycle-gan.git for the Cycle GAN model and at https://github.com/lopes-leonor/DAT-DL-classification.git for the DL classification model.

## Abbreviations

AI: Artificial Intelligence
APS: Atypical Parkinsonian Syndromes
AUC: Area under the ROC curve
CNR: Contrast-to-noise Ratio
DAT: Dopamine Transporter
DL: Deep Learning
FID: Fréchet Inception Distance
FWHM: Full Width at Half Maximum
GANs: Generative Adversarial Networks
Cycle GAN: Cycle-consistency GANs
HPPI: Huashan Parkinsonian PET Imaging
MNI: Montreal Neurological Institute
MSA: Multiple System Atrophy
NC: Normal Controls/Non-parkinsonian Controls
NPV: Negative Predictive Value
PD: Parkinson’s Disease
PET: Positron Emission Tomography
PPMI: Parkinson’s Progression Markers Initiative
PPV: Positive Predictive Value
PSP: Progressive Supranuclear Palsy
RMSE: Root-Mean-Square Error
ROC: Receiver Operating Characteristic
SBR: Striatal Specific Binding Ratio
SD: Standard Deviation
SPECT: Single Photon Emission Computed Tomography
SSIM: Structural Similarity Index Measure
VGA: Visual Grading Analysis
VGAS: VGA Score

## References

[1] Saeed U, Lang AE, Masellis M. Neuroimaging advances in Parkinson’s Disease and atypical parkinsonian syndromes. Front Neurol. 2020;11:572976. 10.3389/fneur.2020.572976. 33178113 10.3389/fneur.2020.572976 PMC7593544

[2] Scherfler C, Schwarz J, Antonini A, et al. Role of DAT-SPECT in the diagnostic work up of parkinsonism. Mov Disord. 2007;22:1229–38. 17486648 10.1002/mds.21505

[3] Benamer TS, Patterson J, Grosset DG, et al. Accurate differentiation of parkinsonism and essential tremor using visual assessment of [123I]-FP-CIT SPECT imaging: the [123I]-FP-CIT study group. Movment Disorders. 2000;15(3):503–10. 10830416

[4] McKeith I, O’Brien J, Walker Z, et al. Sensitivity and specificity of dopamine transporter imaging with ^123^I-FP-CIT SPECT in dementia with Lewy bodies: a phase III, multicenter study. Lancet Neurol. 2007;6:305–13. 17362834 10.1016/S1474-4422(07)70057-1

[5] Booij J, Dubroff J, Pryma D, et al. Diagnostic performance of the Visual Reading of ^123^I-Ioflupane SPECT images with or without quantification in patients with Movement disorders or Dementia. J Nucl Med. 2017;58:1821. 28473597 10.2967/jnumed.116.189266

[6] Palermo G, Giannoni S, Bellini G, Siciliano G, Ceravolo R. Dopamine transporter imaging, current status of a potential biomarker: a Comprehensive Review. Int J Mol Sci. 2021;22. 10.3390/ijms222011234 PMC8538800 34681899

[7] Andringa G, Drukarch B, Bol JGJM, et al. Pinhole SPECT imaging of dopamine transporters correlates with dopamine transporter immunohistochemical analysis in the MPTP mouse model of Parkinson’s disease. NeuroImage. 2005;26:1150–8. 15908232 10.1016/j.neuroimage.2005.03.034

[8] Wenzel M, Milletari F, Krüger J, et al. Automatic classification of dopamine transporter SPECT: deep convolutional neural networks can be trained to be robust with respect to variable image characteristics. Eur J Nucl Med Mol Imaging. 2019;46:2800–11. 31473800 10.1007/s00259-019-04502-5

[9] Hirschauer TJ, Adeli H, Buford JA. Computer-aided diagnosis of Parkinson’s Disease using enhanced probabilistic neural network. J Med Syst. 2015;39:179. 26420585 10.1007/s10916-015-0353-9

[10] Zhao Y, Wu P, Wu J, et al. Decoding the dopamine transporter imaging for the differential diagnosis of parkinsonism using deep learning. Eur J Nucl Med Mol Imaging. 2022;49:2798–811. 35588012 10.1007/s00259-022-05804-x PMC9206631

[11] Morbelli S, Esposito G, Arbizu J, et al. EANM practice guideline/SNMMI procedure standard for dopaminergic imaging in parkinsonian syndromes 1.0. Eur J Nucl Med Mol Imaging. 2020;47:1885–912. 32388612 10.1007/s00259-020-04817-8 PMC7300075

[12] Goodfellow IJ, Pouget-Abadie J, Mirza M et al. Generative Adversarial Networks. arXiv preprint. 2014. 10.48550/arXiv.1406.2661.

[13] Calimeri F, Marzullo A, Stamile C, Terracina G. Biomedical data augmentation using generative adversarial neural networks. In: Lintas, A., Rovetta, S., Verschure, P., Villa, A. (eds) Artificial neural networks and machine learning – ICANN 2017. Lecture Notes in Computer Science, vol 10614. Springer, Cham. 2017. 10.1007/978-3-319-68612-7_71c.

[14] Chuquicusma MJM, Hussein S, Burt J, Bagci U. How to fool radiologists with generative adversarial networks? A visual turing test for lung cancer diagnosis. 2018 IEEE 15th International Symposium on Biomedical Imaging. 2018;240–244.

[15] Shokraei Fard A, Reutens DC, Vegh V. From CNNs to GANs for cross-modality medical image estimation. Comput Biol Med. 2022;146:105556. 10.1016/j.compbiomed.2022.105556. 35504221 10.1016/j.compbiomed.2022.105556

[16] Özbey M, Dalmaz O, Dar SUH et al. Unsupervised Medical Image Translation with Adversarial Diffusion Models. *arXiv* preprint. 2023. arxiv.org/abs/2207.08208v3 10.1109/TMI.2023.3290149 37379177

[17] Islam T, Hafiz S, Jim JR, et al. A systematic review of deep learning data augmentation in medical imaging: recent advances and future research directions. Healthc Analytics. 2024;5:100340. 10.1016/j.health.2024.100340.

[18] Xue S, Guo R, Bohn KP, et al. A cross-scanner and cross-tracer deep learning method for the recovery of standard-dose imaging quality from low-dose PET. Eur J Nucl Med Mol Imaging. 2022;49:1843–56. 34950968 10.1007/s00259-021-05644-1 PMC9015984

[19] Guo R, Xue S, Hu J, et al. Using domain knowledge for robust and generalizable deep learning-based CT-free PET attenuation and scatter correction. Nat Commun. 2022;13:5882. 36202816 10.1038/s41467-022-33562-9 PMC9537165

[20] Zhu JY, Park T, Isola P, Efros AA. Unpaired image-to-image translation using cycle-consistent adversarial networks. arXiv preprint.

[21] Lee KW, Chin RKY. A Comparative Study of COVID-19 CT Image Synthesis using GAN and CycleGAN. 2022 IEEE International Conference on Artificial Intelligence in Engineering and Technology. 2022;1–6.

[22] Gu J, Yang TS, Ye JC, Yang DH. CycleGAN denoising of extreme low-dose cardiac CT using wavelet assisted noise disentanglement. Med Image Anal. 2021;74:102209. 34450466 10.1016/j.media.2021.102209

[23] Zhou L, Schaefferkoetter JD, Tham IWK, Huang G, Yan J. Supervised learning with cyclegan for low-dose FDG PET image denoising. Med Image Anal. 2020;65:101770. 32674043 10.1016/j.media.2020.101770

[24] Kalantar R, Hindocha S, Hunter B, et al. Non-contrast CT synthesis using patch-based cycle-consistent generative adversarial network (Cycle-GAN) for radiomics and deep learning in the era of COVID-19. Sci Rep. 2023;13:10568. 10.1038/s41598-023-36712-1. 37386097 10.1038/s41598-023-36712-1 PMC10310777

[25] Postuma RB, Berg D, Stern M, et al. MDS clinical diagnostic criteria for Parkinson’s disease. Mov Disord. 2015;30:1591–601. 26474316 10.1002/mds.26424

[26] Manera AL, Dadar M, Fonov V, Collins DL. CerebrA, registration and manual label correction of Mindboggle-101 atlas for MNI-ICBM152 template. Sci Data. 2020;7:237. 32669554 10.1038/s41597-020-0557-9 PMC7363886

[27] Månsson LG. Methods for the evaluation of image quality: a review. Radiat Prot Dosimetry. 2000;90:89–99.

[28] Fahmi R, Platsch G, Sadr AB, et al. Single-site (123)I-FP-CIT reference values from individuals with non-degenerative parkinsonism comparison with values from healthy volunteers. Eur J Hybrid Imaging. 2020;4:5. 34191214 10.1186/s41824-020-0074-2 PMC8218096

[29] Tinaz S, Chow C, Kuo PH, et al. Semiquantitative analysis of dopamine transporter scans in patients with Parkinson Disease. Clin Nucl Med. 2018;43(1):e1–7. 29112012 10.1097/RLU.0000000000001885 PMC7257254

[30] Seibyl JP, Marek K, Sheff K, et al. Iodine-123–CIT and Iodine-123-FPCIT SPECT measurement of dopamine transporters in healthy subjects and Parkinson’s patients. J Nucl Med. 1998;39:1500. 9744331

[31] Varrone A, Dickson JC, Tossici-Bolt L, et al. European multicenter database of healthy controls for [123I]FP-CIT SPECT (ENC-DAT): age-related effects, gender differences and evaluation of different methods of analysis. Eur J Nucl Med Mol Imaging. 2013;40:213–27. 23160999 10.1007/s00259-012-2276-8

[32] Tossici-Bolt L, Dickson JC, Sera T, et al. Calibration of gamma camera systems for a multicenter European 123I-FP-CIT SPECT normal database. Eur J Nucl Med Mol Imaging. 2011;38:1529–40. 21468761 10.1007/s00259-011-1801-5

[33] Buchert R, Kluge A, Tossici-Bolt L, et al. Reduction in camera-specific variability in [123I]FP-CIT SPECT outcome measures by image reconstruction optimized for multisite settings: impact on age-dependence of the specific binding ratio in the ENC-DAT database of healthy controls. Eur J Nucl Med Mol Imaging. 2016;43:1323–36. 26816194 10.1007/s00259-016-3309-5

[34] Tang H, Liu H, Xu D, Torr PHS, Sebe N. AttentionGAN: unpaired image-to-image translation using attention-guided generative adversarial networks. IEEE Trans Neural Netw Learn Syst. 2023;34(4):1972–87. 10.1109/TNNLS.2021.3105725. 34473628 10.1109/TNNLS.2021.3105725

[35] Saharia C, Chan W, Chang H et al. Palette: Image-to-Image Diffusion Models. In: ACM SIGGRAPH 2022 Conference Proceedings. SIGGRAPH ’22. Association for Computing Machinery; 2022. 10.1145/3528233.3530757

[36] Ho J, Jain A, Abbeel P. Denoising Diffusion Probabilistic Models. *arXiv [csLG]*. Published online 2020. http://arxiv.org/abs/2006.11239

[37] Takamiya K, Iwamoto Y, Nonaka M, Chen YW. CT Brain Image Synthesization from MRI Brain Images Using CycleGAN. 2023 IEEE International Conference on Consumer Electronics. 2023;1–4.

[38] Hammami M, Friboulet D, Kechichian R. Cycle GAN-Based Data Augmentation For Multi-Organ Detection In CT Images Via Yolo. 2020 IEEE International Conference on Image Processing. 2020;390–393.

[39] Skandarani Y, Jodoin P-M, Lalande A. GANs for medical image synthesis: an empirical study. J Imaging. 2023;9(3):69. 10.3390/jimaging9030069. 36976120 10.3390/jimaging9030069 PMC10055771

[40] Hiasa Y, Otake Y, Takao M, et al. Cross-modality image synthesis from unpaired data using CycleGAN BT - Simulation and Synthesis in Medical Imaging. Springer International Publishing; 2018. pp. 31–41.

[41] Cohen JP, Luck M, Honari S. Distribution matching losses can hallucinate features in medical image translation. arXiv preprint. 2018. 10.48550/arXiv.1805.08841

[42] Shamsolmoali P, Zareapoor M, Granger E, et al. Image synthesis with adversarial networks: a comprehensive survey and case studies. Inform Fusion. 2021;72:126–46.

[43] Joshi A, Koeppe RA, Fessler JA. Reducing between scanner differences in multi-center PET studies. Neuroimage. 2009;46(1):154–159. 10.1016/j.neuroimage.2009.01.057 10.1016/j.neuroimage.2009.01.057 PMC4308413 19457369

