# Supplementary material for: Dopaminergic PET to SPECT domain adaptation: a cycle GAN translation approach
Source: Eur J Nucl Med Mol Imaging. 2024 Nov 19;52(3):851–63. doi: 10.1007/s00259-024-06961-x (PMC11754385; doi:10.1007/s00259-024-06961-x)
Supplement: Supplementary file 1 — Supplementary Material 1 [file 259_2024_6961_MOESM1_ESM.docx]

**Supplementary Materials**

**Huashan Parkinsonian PET Imaging (HPPI) inclusion criteria**

The inclusion criteria for healthy subjects were: (1) no previous history of neurological or psychiatric illness; (2) no prior exposure to neuroleptic agents or drug addiction; (3) normal neurological or MRI examination; (4) no previous use of drugs with DAT blocking components. The inclusion criteria for subjects with normal DAT imaging were:  (1) visited the Movement Disorders Clinic with Parkinson-related complains; (2) parkinsonism was considered clinically at the first visit, and finally was diagnosed as essential tremor, depression or anxiety; (3) DAT PET imaging was determined as normal based on visual assessment by at least two experienced neuroimaging experts from the PET Center and the semi-quantitative value was within the normal range.

**Detailed Cycle GAN Model**

**Model**

A 3D CycleGAN was developed to make the image-to-image translation between two domains, DAT PET imaging and DAT SPECT imaging. The CycleGAN model includes two generators (G_PS_ - PET to SPECT- and G_SP_ – SPECT to PET) and two associated adversarial discriminators (D_P_ and D_S_).

Each one of the discriminators encourages its corresponding generator to synthesize images similar to the original ones by minimizing an adversarial loss function. The latter is defined, for PET to SPECT translation, as:

$\mathcal{L}_{adv}(G_{PS}, D_{S}, P, S)=\mathbb{E}_{S\sim p_{data}\left( S \right)}[\log D_{S}(S)]+\mathbb{E}_{P\sim p_{data}(P)}[\log(1-D_{S}(G_{PS}\left( P \right))]$, (1)

Where P and S are the sampled elements from each probability distribution of PET and SPECT images respectively, G_PS_ is the generator that translates PET to SPECT images and D_S_ the discriminator that distinguishes between real and synthetic SPECT. A similar adversarial loss for SPECT to PET translation, $\mathcal{L}_{adv}(G_{SP}, D_{P}, S, P)$, is used.

The synthesized SPECT images are then translated back to the original PET domain, with the G_SP_ (and vice-versa for the synthetic PET images). The cycle consistency loss, $\mathcal{L}_{cyc}\left( G_{PS}, G_{SP} \right),$ helps ensure that the translated images are similar to the real ones:

$\mathcal{L}_{cyc}\left( G_{PS}, G_{SP} \right)=\mathbb{E}_{P\sim p_{data}\left( P \right)}\left[ \parallel G_{SP}\left( G_{PS}\left( P \right) \right)-P\parallel\right]+\mathbb{E}_{S\sim p_{data}\left( S \right)}\left[ \parallel G_{PS}\left( G_{SP}\left( S \right) \right)-S\parallel\right]$.

The PET scan is inputted into the G_SP_ and vice-versa (SPECT to the G_PS_) and the output image is compared to the real PET (and SPECT) image, through the identity loss:

$\mathcal{L}_{id}\left( G_{PS}, G_{SP} \right)=\mathbb{E}_{P\sim p_{data}\left( P \right)}\left[ \parallel G_{SP}(P)-P\parallel\right]+\mathbb{E}_{S\sim p_{data}\left( S \right)}\left[ \parallel G_{PS}(S)-S\parallel\right]$.

The final and total cycleGAN loss is:

$\mathcal{L}\left( G_{PS}, G_{SP}, D_{P}, D_{S} \right)=\alpha_{1}\mathcal{[L}_{adv}\left( G_{PS}, D_{S}, P, S \right)+\mathcal{L}_{adv}\left( G_{SP}, D_{P}, S, P \right)]+\alpha_{2}\mathcal{L}_{cyc}\left( G_{PS}, G_{SP} \right)+\alpha_{3}\mathcal{L}_{id}\left( G_{PS}, G_{SP} \right)$,

where α_1_, α_2_, α_3_ weight the importance of each loss.

Our generator network consists of an encoder consisting of three downsampling blocks and six residual blocks with residual connections, and a decoder consisting of two upsampling blocks, and a final convolution layer and hyperbolic tangent activation function. The dowsampling blocks include a 3D convolutional layer, instance normalization and a RELU activation function. The upsampling blocks have a 3D transposed convolution layer, followed by instance normalization layer and a RELU activation.

**Synthetic Images processing**

After generation of synthetic SPECT images we applied the SPM brain mask in the MNI space and a Gaussian filter of 1.7 FWHM to smooth the images with a grid-like texture.

The mean images of real PET, real SPECT and synthetic SPECT test datasets are shown in Supplementary Figure 1.

In Supplementary Figure 2, two examples of synthetic SPECT images are displayed to illustrate the grid-like texture of the synthetic SPECT images before application of Gaussian filter.

In Supplementary Figure 3, an example of a synthetic image with artifacts is shown.


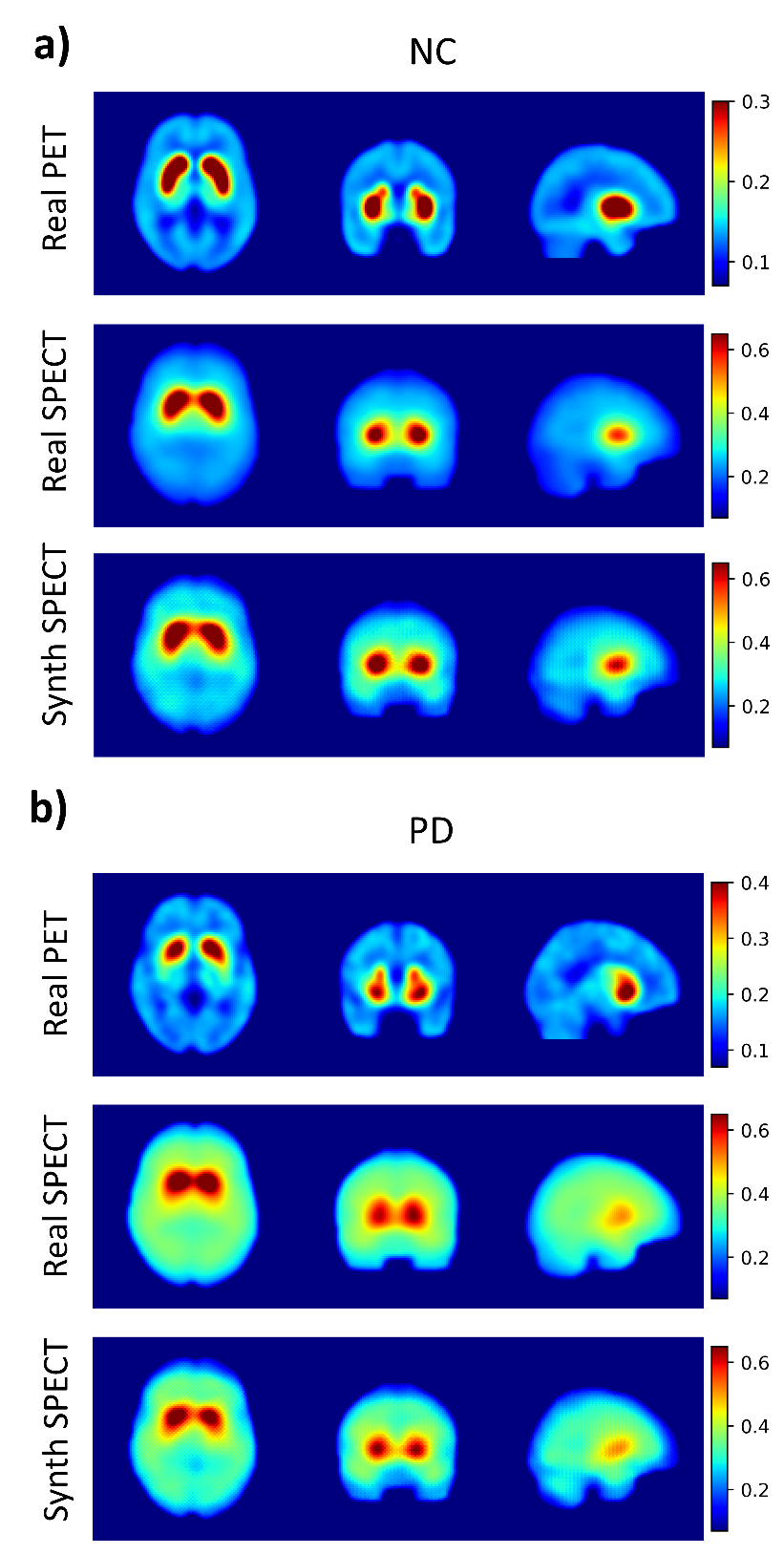


**Supplementary Figure 1. Mean images of real PET (upper row), real SPECT (middle row) and synthetic SPECT (bottom row) test datasets from (a) normal controls and (b) Parkinson’s disease.** NC: Normal controls; PD: Parkinson disease.

**
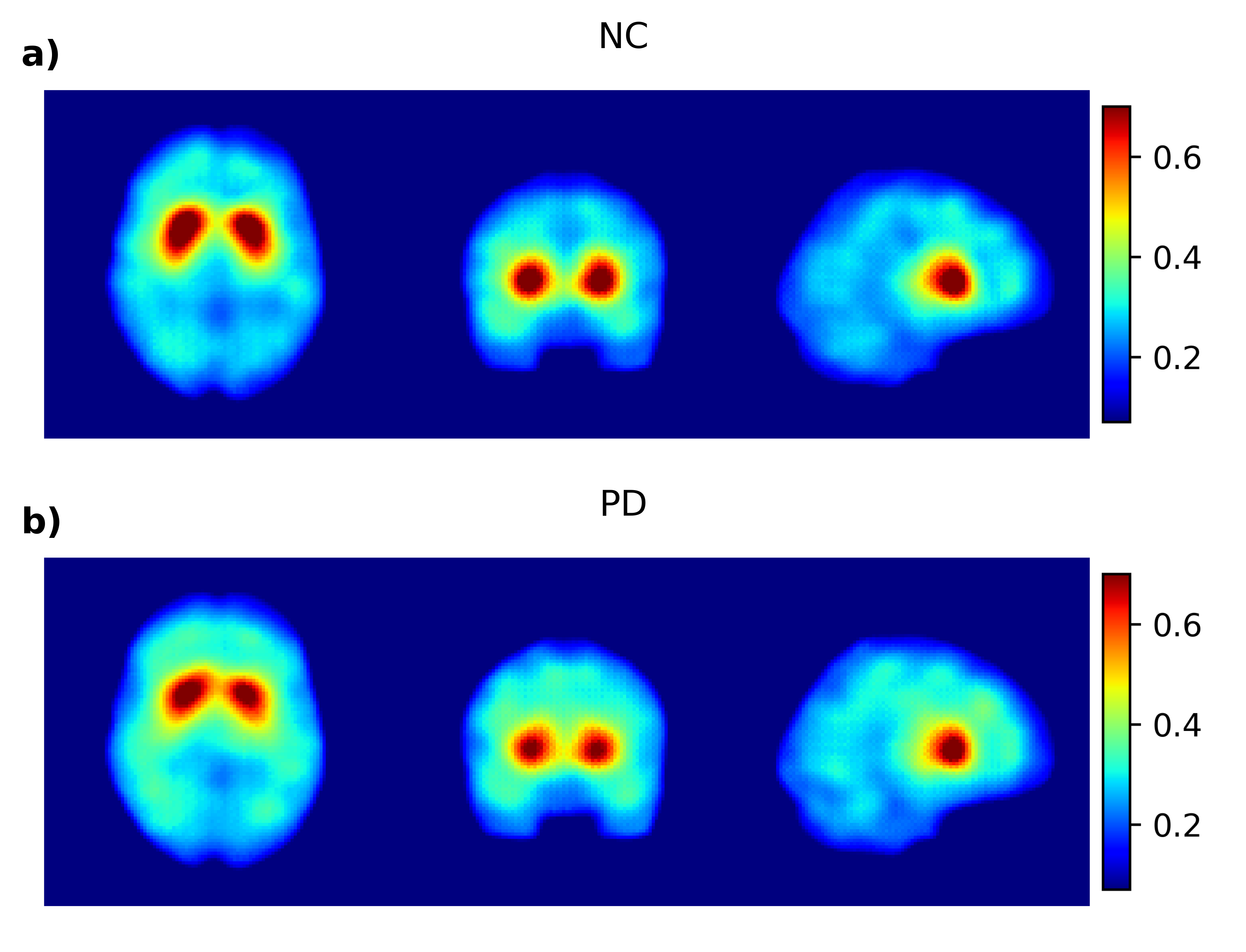
**

**Supplementary Figure 2. Synthetic SPECT images of a) NC and b) PD without Gaussian filter applied.** NC: Normal controls; PD: Parkinson disease.

**
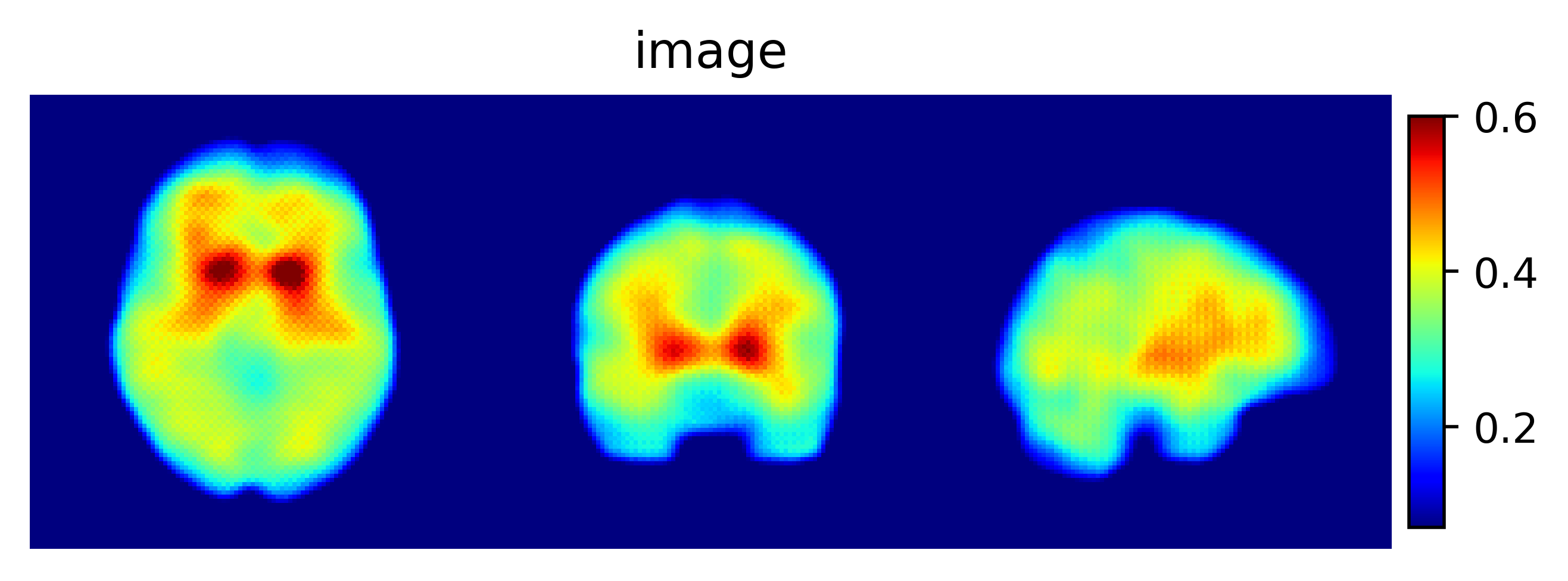
**

**Supplementary Figure 2. Example of Synthetic SPECT image with artifacts.**

**Blind Visual Assessment of individual readers**

In Supplementary Table 1, the scores of each individual reader are presented in the 4 criteria analyzed: synthetic Appearance, level of noise, presence of artifacts and confidence in diagnosis. Scores of both real and synthetic SPECT datasets are presented and compared.

| Reader | Criteria | Real SPECT  (Median ± IQR) | Synthetic SPECT  (Median ± IQR) | P-value |
| --- | --- | --- | --- | --- |
| #1 | Synthetic Appearance | 2.0 ± 1.75 | 2.0 ± 0.0 | 0.912 |
|  | Level of Noise | 2.0 ± 0.75 | 2.0 ± 0.75 | 0.8534 |
|  | Presence of Artifacts | 1.0 ± 0.0 | 1.0 ± 0.0 | 0.73936 |
|  | Confidence in Diagnosis | 2.5 ± 1.0 | 2.0 ± 0.75 | 0.1051 |
| #2 | Synthetic Appearance | 1.5 ± 1.0 | 1.0 ± 0.0 | 0.3527 |
|  | Level of Noise | 1.0 ± 0.0 | 1.0 ± 0.0 | 1.0 |
|  | Presence of Artifacts | 1.0 ± 0.75 | 1.0 ± 0.75 | 0.9705 |
|  | Confidence in Diagnosis | 2.0 ± 0.75 | 2.0 ± 1.5 | 0.8534 |
| #3 | Synthetic Appearance | 2.0 ± 0.0 | 2.0 ± 0.0 | 1.0 |
|  | Level of Noise | 1.0 ± 0.0 | 1.0 ± 0.0 | 1.0 |
|  | Presence of Artifacts | 2.0 ± 0.0 | 2.0 ± 0.0 | 1.0 |
|  | Confidence in Diagnosis | 3.0 ± 0.75 | 3.0 ± 0.75 | 1.0 |
| #4 | Synthetic Appearance | 3.0 ± 2.0 | 1.0 ± 1.5 | 0.27986 |
|  | Level of Noise | 2.0 ± 0.75 | 2.0 ± 1.0 | 0.4359 |
|  | Presence of Artifacts | 3.0 ± 2.0 | 1.5 ± 1.75 | 0.4359 |
|  | Confidence in Diagnosis | 3.0 ± 1.0 | 2.0 ± 1.5 | 0.21756 |

**Supplementary Table 1. Synthetic Appearance, level of noise, presence of artifacts and confidence in diagnosis scores per dataset (Real and Synthetic SPECT images) of each individual reader.** Values are presented as median score of all images ± interquartile range (IQR). P-values of the differences between Real and Synthetic SPECT datasets are also presented as obtained by Mann-Whitney U test.

**PPMI Sponsor and funding Partners**

PPMI is sponsored and partially funded by The Michael J. Fox Foundation for Parkinson’s Research (MJFF). Other funding partners include a consortium of industry players, non-profit organizations and private individuals.

Funding Partners

1. 4D Pharma

2. AbbVie Inc.

3. AcureX Therapeutics

4. Allergan

5. Amathus Therapeutics

6. Aligning Science Across Parkinson’s (ASAP)

7. Avid Radiopharmaceuticals

8. Bial Biotech

9. Biogen

10. BioLegend

11. BlueRock Therapeutics

12. Bristol Myers Squibb

13. Calico Life Sciences LLC

14. Celgene Corporation

15. DaCapo Brainscience

16. Denali Therapeutics

17. The Edmond J. Safra Foundation

18. Eli Lilly and Company

19. Gain Therapeutics

20. GE Healthcare

21. GlaxoSmithKline

22. Golub Capital

23. Handl Therapeutics

24. Insitro

25. Janssen Pharmaceuticals

26. Lundbeck

27. Merck & Co., Inc.

28. Meso Scale Diagnostics, LLC

29. Neurocrine Biosciences

30. Pfizer Inc.

31. Piramal Imaging

32. Prevail Therapeutics

33. F. Hoffmann-La Roche Ltd and its affiliated company Genentech Inc.

34. Sanofi Genzyme

35. Servier

36. Takeda Pharmaceutical Company

37. Teva Neuroscience, Inc.

38. UCB

39. Vanqua Bio

40. Verily Life Sciences

41. Voyager Therapeutics, Inc.

42. Yumanity Therapeutics, Inc.
